# Supplementary material for: Enhanced Cas12a multi-gene regulation using a CRISPR array separator
Source: eLife. 2021 Sep 9;10:e66406. doi: 10.7554/eLife.66406 (PMC8478413; doi:10.7554/eLife.66406)
Supplement: Supplementary file 4. [file elife-66406-supp4.docx]

CAGp-FireflyLuciferase-Array-Terminator (Fig. 3C)

CAG promoter

Firefly luciferase

CRISPR array (AAAT separator, Repeat, 50%-GC nonsense spacer and GFP-targeting spacer)

SV40 terminator

GTGAGCCCCACGTTCTGCTTCACTCTCCCCATCTCCCCCCCCTCCCCACCCCCAATTTTGTATTTATTTATTTTTTAATTATTTTGTGCAGCGATGGGGGCGGGGGGGGGGGGGGCGCGCGCCAGGCGGGGCGGGGCGGGGCGAGGGGCGGGGCGGGGCGAGGCGGAGAGGTGCGGCGGCAGCCAATCAGAGCGGCGCGCTCCGAAAGTTTCCTTTTATGGCGAGGCGGCGGCGGCGGCGGCCCTATAAAAAGCGAAGCGCGCGGCGGGCGGGAGTCGCTGCGTTGCCTTCGCCCCGTGCCCCGCTCCGCGCCGCCTCGCGCCGCCCGCCCCGGCTCTGACTGACCGCGTTACTCCCACAGGTGAGCGGGCGGGACGGCCCTTCTCCTCCGGGCTGTAATTAGCGCTTGGTTTAATGACGGCTCGTTTCTTTTCTGTGGCTGCGTGAAAGCCTTAAAGGGCTCCGGGAGGGCCCTTTGTGCGGGGGGGAGCGGCTCGGGGGGTGCGTGCGTGTGTGTGTGCGTGGGGAGCGCCGCGTGCGGCCCGCGCTGCCCGGCGGCTGTGAGCGCTGCGGGCGCGGCGCGGGGCTTTGTGCGCTCCGCGTGTGCGCGAGGGGAGCGCGGCCGGGGGCGGTGCCCCGCGGTGCGGGGGGGCTGCGAGGGGAACAAAGGCTGCGTGCGGGGTGTGTGCGTGGGGGGGTGAGCAGGGGGTGTGGGCGCGGCGGTCGGGCTGTAACCCCCCCCTGCACCCCCCTCCCCGAGTTGCTGAGCACGGCCCGGCTTCGGGTGCGGGGCTCCGTGCGGGGCGTGGCGCGGGGCTCGCCGTGCCGGGCGGGGGGTGGCGGCAGGTGGGGGTGCCGGGCGGGGCGGGGCCGCCTCGGGCCGGGGAGGGCTCGGGGGAGGGGCGCGGCGGCCCCGGAGCGCCGGCGGCTGTCGAGGCGCGGCGAGCCGCAGCCATTGCCTTTTATGGTAATCGTGCGAGAGGGCGCAGGGACTTCCTTTGTCCCAAATCTGGCGGAGCCGAAATCTGGGAGGCGCCGCCGCACCCCCTCTAGCGGGCGCGGGCGAAGCGGTGCGGCGCCGGCAGGAAGGAAATGGGCGGGGAGGGCCTTCGTGCGTCGCCGCGCCGCCGTCCCCTTCTCCATCTCCAGCCTCGGGGCTGCCGCAGGGGGACGGCTGCCTTCGGGGGGGACGGGGCAGGGCGGGGTTCGGCTTCTGGCGTGTGACCGGCGGCTCTAGAGCCTCTGCTAACCATGTTCATGCCTTCTTCTTTTTCCTACAGCTCCTGGGCAACGTGCTGGTTGTTGTGCTGTCTCATCATTTTGGCAAAGAATTCACCATGGAAGACGCCAAAAACATAAAGAAAGGCCCGGCGCCATTCTATCCGCTAGAGGATGGAACCGCTGGAGAGCAACTGCATAAGGCTATGAAGAGATACGCCCTGGTTCCTGGAACAATTGCTTTTACAGATGCACATATCGAGGTGAACATCACGTACGCGGAATACTTCGAAATGTCCGTTCGGTTGGCAGAAGCTATGAAACGATATGGGCTGAATACAAATCACAGAATCGTCGTATGCAGTGAAAACTCTCTTCAATTCTTTATGCCGGTGTTGGGCGCGTTATTTATCGGAGTTGCAGTTGCGCCCGCGAACGACATTTATAATGAACGTGAATTGCTCAACAGTATGAACATTTCGCAGCCTACCGTAGTGTTTGTTTCCAAAAAGGGGTTGCAAAAAATTTTGAACGTGCAAAAAAAATTACCAATAATCCAGAAAATTATTATCATGGATTCTAAAACGGATTACCAGGGATTTCAGTCGATGTACACGTTCGTCACATCTCATCTACCTCCCGGTTTTAATGAATACGATTTTGTACCAGAGTCCTTTGATCGTGACAAAACAATTGCACTGATAATGAACTCCTCTGGATCTACTGGGTTACCTAAGGGTGTGGCCCTTCCGCATAGAACTGCCTGCGTCAGATTCTCGCATGCCAGAGATCCTATTTTTGGCAATCAAATCATTCCGGATACTGCGATTTTAAGTGTTGTTCCATTCCATCACGGTTTTGGAATGTTTACTACACTCGGATATTTGATATGTGGATTTCGAGTCGTCTTAATGTATAGATTTGAAGAAGAGCTGTTTTTACGATCCCTTCAGGATTACAAAATTCAAAGTGCGTTGCTAGTACCAACCCTATTTTCATTCTTCGCCAAAAGCACTCTGATTGACAAATACGATTTATCTAATTTACACGAAATTGCTTCTGGGGGCGCACCTCTTTCGAAAGAAGTCGGGGAGGCGGTTGCAAAACGCTTCCATCTTCCAGGGATACGACAAGGATATGGGCTCACTGAGACTACATCAGCTATTCTGATTACACCCGAGGGGGATGATAAACCGGGCGCGGTCGGTAAAGTTGTTCCATTTTTTGAAGCGAAGGTTGTGGATCTGGATACCGGGAAAACGCTGGGCGTTAATCAGAGAGGCGAATTATGTGTCAGAGGACCTATGATTATGTCCGGTTATGTAAACAATCCGGAAGCGACCAACGCCTTGATTGACAAGGATGGATGGCTACATTCTGGAGACATAGCTTACTGGGACGAAGACGAACACTTCTTCATAGTTGACCGCTTGAAGTCTTTAATTAAATACAAAGGATACCAGGTGGCCCCCGCTGAATTGGAGTCGATATTGTTACAACACCCCAACATCTTCGACGCGGGCGTGGCAGGTCTTCCCGACGATGACGCCGGTGAACTTCCCGCCGCCGTTGTTGTTTTGGAGCACGGAAAGACGATGACGGAAAAAGAGATCGTGGATTACGTCGCCAGTCAAGTAACAACCGCGAAAAAGTTGCGCGGAGGAGTTGTGTTTGTGGACGAAGTACCGAAAGGTCTTACCGGAAAACTCGACGCAAGAAAAATCAGAGAGATCCTCATAAAGGCCAAGAAGGGCGGAAAGTCCAAATTGTAAGGATCCTTTCTTTCTTTAAATAATTTCTACTAAGTGTAGATAATGCCTAGACCTGTTGGGAAAATAATTTCTACTAAGTGTAGATCTCCCTATCAGTGATAGAGAACAAATAATTTCTACTAAGTGTAGATCGAGTTTTCTTTTTGTTTTGTACAAGTAAGTTAACTTGTTTATTGCAGCTTATAATGGTTACAAATAAAGCAATAGCATCACAAATTTCACAAATAAAGCATTTTTTTCACTGCATTCTAGTTGTGGTTTGTCCAAACTCATCAATGTATCTTATCATGTCTGGATC

CAGp-BFP-Triplex-Leader-Array-Terminator (Fig. 5)

CAG promoter

BFP

Triplex

Lachnospiraceae bacterium leader sequence

CRISPR array (AAAT separator, Repeat)

SV40 terminator

GTGAGCCCCACGTTCTGCTTCACTCTCCCCATCTCCCCCCCCTCCCCACCCCCAATTTTGTATTTATTTATTTTTTAATTATTTTGTGCAGCGATGGGGGCGGGGGGGGGGGGGGCGCGCGCCAGGCGGGGCGGGGCGGGGCGAGGGGCGGGGCGGGGCGAGGCGGAGAGGTGCGGCGGCAGCCAATCAGAGCGGCGCGCTCCGAAAGTTTCCTTTTATGGCGAGGCGGCGGCGGCGGCGGCCCTATAAAAAGCGAAGCGCGCGGCGGGCGGGAGTCGCTGCGTTGCCTTCGCCCCGTGCCCCGCTCCGCGCCGCCTCGCGCCGCCCGCCCCGGCTCTGACTGACCGCGTTACTCCCACAGGTGAGCGGGCGGGACGGCCCTTCTCCTCCGGGCTGTAATTAGCGCTTGGTTTAATGACGGCTCGTTTCTTTTCTGTGGCTGCGTGAAAGCCTTAAAGGGCTCCGGGAGGGCCCTTTGTGCGGGGGGGAGCGGCTCGGGGGGTGCGTGCGTGTGTGTGTGCGTGGGGAGCGCCGCGTGCGGCCCGCGCTGCCCGGCGGCTGTGAGCGCTGCGGGCGCGGCGCGGGGCTTTGTGCGCTCCGCGTGTGCGCGAGGGGAGCGCGGCCGGGGGCGGTGCCCCGCGGTGCGGGGGGGCTGCGAGGGGAACAAAGGCTGCGTGCGGGGTGTGTGCGTGGGGGGGTGAGCAGGGGGTGTGGGCGCGGCGGTCGGGCTGTAACCCCCCCCTGCACCCCCCTCCCCGAGTTGCTGAGCACGGCCCGGCTTCGGGTGCGGGGCTCCGTGCGGGGCGTGGCGCGGGGCTCGCCGTGCCGGGCGGGGGGTGGCGGCAGGTGGGGGTGCCGGGCGGGGCGGGGCCGCCTCGGGCCGGGGAGGGCTCGGGGGAGGGGCGCGGCGGCCCCGGAGCGCCGGCGGCTGTCGAGGCGCGGCGAGCCGCAGCCATTGCCTTTTATGGTAATCGTGCGAGAGGGCGCAGGGACTTCCTTTGTCCCAAATCTGGCGGAGCCGAAATCTGGGAGGCGCCGCCGCACCCCCTCTAGCGGGCGCGGGCGAAGCGGTGCGGCGCCGGCAGGAAGGAAATGGGCGGGGAGGGCCTTCGTGCGTCGCCGCGCCGCCGTCCCCTTCTCCATCTCCAGCCTCGGGGCTGCCGCAGGGGGACGGCTGCCTTCGGGGGGGACGGGGCAGGGCGGGGTTCGGCTTCTGGCGTGTGACCGGCGGCTCTAGAGCCTCTGCTAACCATGTTCATGCCTTCTTCTTTTTCCTACAGCTCCTGGGCAACGTGCTGGTTGTTGTGCTGTCTCATCATTTTGGCAAAGAATTGAATTCGTCGCCACCATGGAGCTGATTAAGGAGAACATGCACATGAAGCTGTACATGGAGGGCACCGTGGACAACCATCACTTCAAGTGCACATCCGAGGGCGAAGGCAAGCCCTACGAGGGCACCCAGACCATGAGAATCAAGGTGGTCGAGGGCGGCCCTCTCCCCTTCGCCTTCGACATCCTGGCTACTAGCTTCCTCTACGGCAGCAAGACCTTCATCAACCACACCCAGGGCATCCCCGACTTCTTCAAGCAGTCCTTCCCTGAGGGCTTCACATGGGAGAGAGTCACCACATACGAAGACGGGGGCGTGCTGACCGCTACCCAGGACACCAGCCTCCAGGACGGCTGCCTCATCTACAACGTCAAGATCAGAGGGGTGAACTTCACATCCAACGGCCCTGTGATGCAGAAGAAAACACTCGGCTGGGAGGCCTTCACCGAGACGCTGTACCCCGCTGACGGCGGCCTGGAAGGCAGAAACGACATGGCCCTGAAGCTCGTGGGCGGGAGCCATCTGATCGCAAACATCAAGACCACATATAGATCCAAGAAACCCGCTAAGAACCTCAAGATGCCTGGCGTCTACTATGTGGACTACAGACTGGAAAGAATCAAGGAGGCCAACAACGAGACCTACGTCGAGCAGCACGAGGTGGCAGTGGCCAGATACTGCGACCTCCCTAGCAAACTGGGGCACAAGCTTAATTAAGAATTCGGATCACGCGTGATTCGTCAGTAGGGTTGTAAAGGTTTTTCTTTTCCTGAGAAAACAACCTTTTGTTTTCTCAGGTTTTGCTTTTTGGCCTTTCCCTAGCTTTAAAAAAAAAAAAGCAAAAGGTACCTGATTTAATTGCAAATCTTTGAAATAATGCAGACTTAAATTTATAAATTCATGGAATAAGGTGATTTTATTGTGAAAAAATACTCGTATTTTGTTGGAAAAACATCTTTTTGTTGTATAATATGATGATATACGGGATCCTTTCTTTCTTTAAATAATTTCTACTAAGTGTAGATAAAAGTGCCACTCCTTAGGGAAATAATTTCTACTAAGTGTAGATAGATGAGATGGTGACAGATAAAATAATTTCTACTAAGTGTAGATCTCCAGAGCCCGACTCGCCGAAATAATTTCTACTAAGTGTAGATTCGGCTTGCGGGGAGACTTCAAATAATTTCTACTAAGTGTAGATCACCCTTCGGCCCGCCACCCAAATAATTTCTACTAAGTGTAGATAGAAAGGGAATCCCAGGGCCAAATAATTTCTACTAAGTGTAGATCTCCCTATCAGTGATAGAGAAAATAATTTCTACTAAGTGTAGATGTTAACTTGTTTATTGCAGCTTATAATGGTTACAAATAAAGCAATAGCATCACAAATTTCACAAATAAAGCATTTTTTTCACTGCATTCTAGTTGTGGTTTGTCCAAACTCATCAATGTATCTTATCATGTCTGGATC

4-crRNA arrays where the GFP-targeting spacer occupies different positions (Fig. S3D)

Repeat

GFP-targeting spacer

Dummy spacer 1 (30% GC)

Dummy spacer 2 (30% GC)

Dummy spacer 3 (30% GC)

Position 1/4

AATTTCTACTAAGTGTAGATctccctatcagtgatagagaacAATTTCTACTAAGTGTAGATATTCACTATAAGTTGGTGTTaatttctactaagtgtagatGAAATTGGCTATCCAAGTAAAAATTTCTACTAAGTGTAGATGCATCTATTCATAAGAAGTTAATTTCTACTAAGTGTAGAT

Position 2/4

AATTTCTACTAAGTGTAGATGAAATTGGCTATCCAAGTAAAAATTTCTACTAAGTGTAGATctccctatcagtgatagagaacaatttctactaagtgtagatGCATCTATTCATAAGAAGTT AATTTCTACTAAGTGTAGATATTCACTATAAGTTGGTGTTAATTTCTACTAAGTGTAGAT

Position 3/4

AATTTCTACTAAGTGTAGATGCATCTATTCATAAGAAGTTAATTTCTACTAAGTGTAGATGAAATTGGCTATCCAAGTAAAaatttctactaagtgtagatctccctatcagtgatagagaacAATTTCTACTAAGTGTAGATATTCACTATAAGTTGGTGTTAATTTCTACTAAGTGTAGAT

Position 4/4

AATTTCTACTAAGTGTAGATATTCACTATAAGTTGGTGTTAATTTCTACTAAGTGTAGATGCATCTATTCATAAGAAGTTaatttctactaagtgtagatGAAATTGGCTATCCAAGTAAAAATTTCTACTAAGTGTAGATctccctatcagtgatagagaacAATTTCTACTAAGTGTAGAT

In vitro-transcribed CRISPR arrays used for Cas12a cleavage assay (Figs. 4, S4)

70%-GC dummy spacer; no synSeparator

Upstream sequence

Dummy gRNA (70%-GC spacer)

GFP-targeting gRNA

Downstream sequence

GGATCCTCATAAAGGCCAAGAAGGGCGGAAAGTCCAAATTGTAAGGATCCTTTCTTTAATTTCTACTAAGTGTAGATCGCCAAACGTGCCCTGACGGGAATTTCTACTAAGTGTAGATCTCCCTATCAGTGATAGAGAACAATTTCTACTAAGTGTAGATGTTAACTTGTTTATTGCAGCTTATAATGGTTACAAATAAAGCAATAGCATCACAAATTTCACAAATAAAGCATTTTTTTCACTGCATTCTAGTTGTGGTTTGTCCAAACTCATCAATGTATCTTATCATGTCTGGATC

70%-GC dummy spacer; AAAT synSeparator

Upstream sequence + synSeparator

Dummy gRNA + synSeparator (70%-GC spacer)

GFP-targeting gRNA + synSeparator

Downstream sequence

GGATCCTCATAAAGGCCAAGAAGGGCGGAAAGTCCAAATTGTAAGGATCCTTTCTTTAAATAATTTCTACTAAGTGTAGATCGCCAAACGTGCCCTGACGGGAAATAATTTCTACTAAGTGTAGATCTCCCTATCAGTGATAGAGAACAAATAATTTCTACTAAGTGTAGATGTTAACTTGTTTATTGCAGCTTATAATGGTTACAAATAAAGCAATAGCATCACAAATTTCACAAATAAAGCATTTTTTTCACTGCATTCTAGTTGTGGTTTGTCCAAACTCATCAATGTATCTTATCATGTCTGGATC

30%-GC dummy spacer; no synSeparator

Upstream sequence

Dummy gRNA (30%-GC spacer)

GFP-targeting gRNA

Downstream sequence

GGATCCTCATAAAGGCCAAGAAGGGCGGAAAGTCCAAATTGTAAGGATCCTTTCTTTCTTTAATTTCTACTAAGTGTAGATATTCACTATAAGTTGGTGTTAATTTCTACTAAGTGTAGATCTCCCTATCAGTGATAGAGAACAATTTCTACTAAGTGTAGATCGAGTTTTCTTTTTGTTTTGTACAAGTAAGTTAACTTGTTTATTGCAGCTTATAATGGTTACAAATAAAGCAATAGCATCACAAATTTCACAAATAAAGCATTTTTTTCACTGCATTCTAGTTGTGGTTTGTCCAAACTCATCAATGTATCTTATCATGTCTGGATC
